# Supplementary material for: Splice-Junction-Based Mapping of Alternative Isoforms in the Human Proteome
Source: Cell Rep. Author manuscript; Available in PMC 2020 Jan 15. (PMC6961840; doi:10.1016/j.celrep.2019.11.026)
Supplement: 2 [file NIHMS1546469-supplement-2.zip › DF1/PXD010154/Pancreas/Pancreas_9_MINDY3_LYSLNNCILLK.pdf]

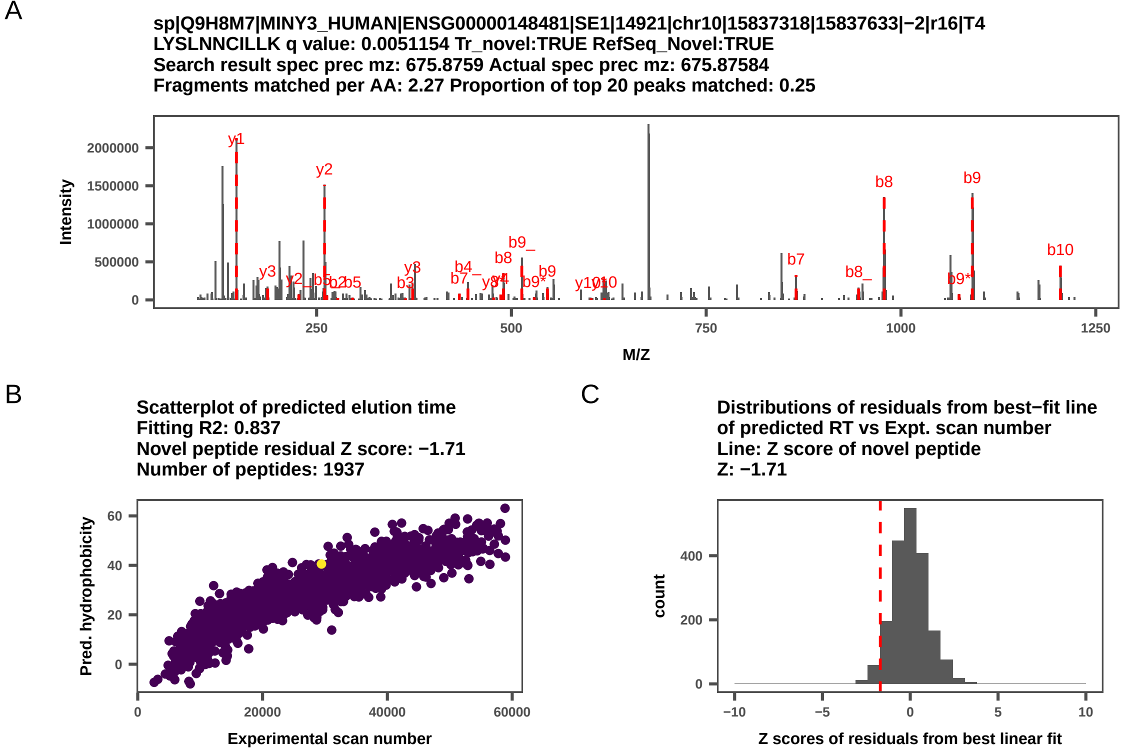
A
sp|Q9H8M7|MINY3_HUMAN|ENSG00000148481|SE1|14921|chr10|15837318|15837633|−2|r16|T4
LYSLNNCILLK q value: 0.0051154 Tr_novel:TRUE RefSeq_Novel:TRUE
Search result spec prec mz: 675.8759 Actual spec prec mz: 675.87584
Fragments matched per AA: 2.27 Proportion of top 20 peaks matched: 0.25
Intensity
2000000
1500000
1000000
500000
0
y1
y2
y3
b3
b4
b7
b8
b9
b10
M/Z
250
500
750
1000
1250
B
Scatterplot of predicted elution time
Fitting R2: 0.837
Novel peptide residual Z score: −1.71
Number of peptides: 1937
Pred. hydrophobicity
60
40
20
0
Experimental scan number
0
20000
40000
60000
C
Distributions of residuals from best−fit line
of predicted RT vs Expt. scan number
Line: Z score of novel peptide
Z: −1.71
count
400
200
0
Z scores of residuals from best linear fit
−10
−5
0
5
10
